# Development and Validation of a Prognostic Risk Score for Patients With Cancer and Neutropenic Fever Presenting to the Emergency Department

**DOI:** 10.1016/j.acepjo.2026.100347

**Published:** 2026-02-26

**Authors:** Sandy Nath, Aiham Qdaisat, Andriy Derkach, Afia Babar, Deepti Zalavadia, Patrick Chaftari, Ziyi Li, Monica K. Wattana, Joseph Schmeltz, Rocio Perez Johnston, Eduardo Ortiz, Kumar Alagappan, Adam Klotz, Sai-Ching Jim Yeung

**Affiliations:** 1 Emergency Care Service, Department of Medicine, Memorial Sloan Kettering Cancer Center, New York, New York, USA; 2 Department of Emergency Medicine, The University of Texas MD Anderson Cancer Center, Houston, Texas, USA; 3 Department of Biostatistics, Memorial Sloan Kettering Cancer Center, New York, New York, USA; 4 Department of Employee Health, Memorial Sloan Kettering Cancer Center, New York, New York, USA; 5 Department of Biostatistics, The University of Texas MD Anderson Cancer Center, Houston, Texas, USA; 6 Department of Data and Analytics, Memorial Sloan Kettering Cancer Center, New York, New York, USA; 7 Department of Radiology, Memorial Sloan Kettering Cancer Center, New York, New York, USA; 8 Department of Radiology, University of Colorado Anschutz Medical Campus, Aurora, Colorado, USA

**Keywords:** neutropenic fever, cancer, emergency, score, neutropenia, outcome, prognostic, fever

## Abstract

**Objectives:**

Neutropenic fever (NF) is a life-threatening oncologic emergency associated with significant morbidity and mortality. Current predictive risk-stratification scores’ limitations include outdated criteria, subjectivity, and low usage in the emergency department (ED). We sought to develop and externally validate a pragmatic, objective risk score to predict adverse outcomes in patients presenting to the ED with cancer and NF.

**Methods:**

A retrospective cohort study was performed using data from 2 comprehensive cancer centers. The derivation cohort included adults presenting with NF to Memorial Sloan Kettering Cancer Center. External validation was conducted using data from the MD Anderson Cancer Center. The primary composite adverse outcome included intensive care unit, bacteremia, oxygen supplementation therapy, hospital stay >3 days, and in-hospital mortality. Predictors were assigned points based on the final model coefficients.

**Results:**

The derivation and validation cohorts included 827 and 777 patients, respectively. The final score that included multiple clinical and laboratory biomarkers demonstrated an area under the curve of the receiver operating characteristic curve value of 0.77 (95% CI, 0.73-0.80) in the derivation cohort and 0.78 (95% CI, 0.74-0.83) in the validation cohort. In patients classified as low risk, in-hospital mortality was 0.0%, and intensive care unit admission occurred in < 2% of cases across both cohorts.

**Conclusion:**

The newly developed risk score effectively stratified patients with NF in the ED. Its reliance on readily available, non-subjective data supports the feasibility of its real-time implementation and could improve decision-making and reduce unnecessary hospitalization. Prospective validation in diverse clinical settings is needed.

**The Bottom Line**

Emergency physicians lack a simple, objective way to identify patients with cancer with fever and neutropenia who can be safely discharged. Most are admitted, straining an already overburdened system. In >1600 emergency department visits at 2 leading cancer centers, we developed and validated a simple tool that accurately predicted outcomes. Low-risk patients had no in-hospital deaths, and <2% required intensive care unit level care. This system may streamline clinical decisions, reduce unnecessary admissions, and standardize care.

## 1 Introduction

### 1.1 Background

Lethal infections in the setting of malignancy were first noted by Virchow and contemporaries in the mid-19th century.^1^ One hundred years later, Bodey et al^2^ demonstrated improved clinical outcomes for patients with cancer having suppressed immunity by administering antibiotics. These findings formed the basis for the widespread practice of intravenous (IV) antibiotic administration and inpatient admission for patients presenting with both fever and neutropenia.^3^^,^^4^

Neutropenic fever (NF), which occurs in patients with certain malignancies or following the administration of cytotoxic antineoplastic therapies, has been recognized as an oncologic emergency requiring immediate evaluation and management.^5^ Furthermore, NF represents a significant burden on the US health care system, accounting for 5.2% of all adult oncologic admissions at a cost of $2.3 billion in the United States as of 2012.^6^

### 1.2 Importance

Multiple risk-stratification tools have been developed to identify low-risk populations with NF who may not need hospitalization. “Talcott’s rules,” first published in 1988, identified patients at “low risk for serious medical complications” (Table 1).^7^ Outpatient management was recommended for this subgroup. The Multinational Association of Supportive Care in Cancer (MASCC) score was developed and prospectively validated in 2000 to similarly define a population at low risk for medical complications (Table 2),^8^ suggesting the use of outpatient management for this subgroup. This was further refined for patients with solid tumors who have “stable vital signs” but not “extensive infections” in the Clinical Index of Stable Febrile Neutropenia (CISNE).^9^ Unfortunately, the use of these scoring systems and the adherence to their risk-specific recommendations is limited. A recent study has demonstrated near-universal admission for those presenting to the emergency department (ED) with fever and neutropenia.^10^ Unnecessary admissions strain the health care system, adding to the burden of ED overcrowding. The resulting prolonged boarding times decrease patient satisfaction,^11^ delay care delivery, and strain health-care resources. Furthermore, the MASCC score is limited by low sensitivity in identifying high-risk patients^12^, ^13^, ^14^ and poor negative predictive value^13^; whereas, the CISNE score is limited by low specificity in identifying high-risk patients.^12^^,^^15^ In practical terms, the challenging aspects of bedside application of these scores are their reliance on subjective criteria, complex formulas,^16^ and results that are insufficiently accurate.^10^ In fact, both MASCC and CISNE scores are rarely used in clinical practice in the emergency setting, leading to persistently high ED admission rates for those presenting with NF.^10^^,^^15^^,^^17^

**Table 1:** Characteristics of the patients with cancer and NF presenting to the ED in the MSK (derivation) and MD Anderson (validation) cohorts.

Characteristic	No. of patients (%)		*P*
	MSK (n = 827)	MD Anderson (n = 777)	
Median age, y (IQR)	59 (46-68)	55 (36-68)	< .001
Sex, n (%)			.341
Female	406 (49.1)	363 (46.7)	
Male	421 (50.9)	414 (53.3)	
Median CCI (IQR)	4 (2-6)	7 (6-8)	< .001
Race, n (%)			.019
Asian	90 (10.9)	66 (8.5)	
Black	80 (9.7)	86 (11.1)	
White	569 (68.8)	508 (65.4)	
Others or unknown	88 (10.6)	117 (15.1)	
Cancer type, n (%)			.104
Hematologic	479 (57.9)	481 (61.9)	
Solid tumor	348 (42.1)	296 (38.1)	
Acuity/ESI, n (%)			.012^a^
Emergent	440 (53.2)	440 (56.6)	
Urgent	375 (45.3)	334 (43.0)	
Resuscitation	11 (1.3)	1 (0.1)	
Unknown	1 (0.1)	2 (0.3)	

CCI, Charlson comorbidity index; ED, emergency department; ESI, emergency severity index; MSK, Memorial Sloan Kettering Cancer Center; NF, neutropenic fever.

a Fisher’s exact test.

**Table 2:** Main outcomes of patients with cancer and NF presenting to the ED in the MSK (derivation) and MD Anderson (validation) cohorts.

Characteristic	No. of patients (%)		*P*
	MSK (n = 827)	MD Anderson (n = 777)	
In-hospital mortality, n (%)			.006
No	775 (93.7)	751 (96.7)	
Yes	52 (6.3)	26 (3.3)	
ICU admission, n (%)			.748
No	728 (88.0)	688 (88.5)	
Yes	99 (12.0)	89 (11.5)	
Oxygen supplementation during hospitalization, n (%)			< .001
No	600 (72.6)	451 (58.0)	
Yes	227 (27.4)	326 (42.0)	
Bacteremia, n (%)			.012
No	650 (78.6)	569 (73.2)	
Yes	177 (21.4)	208 (26.8)	
Hospital stay >3 d, N (%)			.049
No	218 (26.4)	172 (22.1)	
Yes	609 (73.6)	605 (77.9)	
Composite adverse outcome, n (%)			.006
No	194 (23.5)	139 (17.9)	
Yes	633 (76.5)	638 (82.1)	

ICU, intensive care unit; ED, emergency department; ESI, emergency severity index; MSK, Memorial Sloan Kettering Cancer Center; NF, neutropenic fever.

Another important reason a new risk scoring system for NF is needed is the change in the composition of patients with NF in the ED who have hematologic malignancies vs solid tumors. Antineoplastic therapies have significantly transformed over the years. Older risk scores were developed in the era when cytotoxic chemotherapy was the principal antineoplastic therapy. In recent years, the implementation of practice guidelines for the use of prophylactic granulocyte colony-stimulating factors has significantly reduced the incidence of neutropenia in patients with solid tumors. As a result of advances in targeted therapies, a decrease in reliance on cytotoxic chemotherapy has also decreased the risk of clinically significant neutropenia. Overall, the composition of patients with NF presenting to the ED has shifted, with an increasing proportion of them having hematologic malignancies.^18^^,^^19^

### 1.3 Goals of This Investigation

In this study, we aimed to create an objective risk score for adverse outcomes in patients with cancer with NF in the ED. The goal is to develop and externally validate a new risk score to identify low-risk patients with NF in the ED who may be considered for outpatient management. We created the scoring system using objective measures as variables (avoiding variations from subjective assessments by ED clinicians) and data readily available to the general ED. We also designed the new risk score to predict the risk of both major and minor inpatient complications so that these high-risk patients can be admitted for treatment of NF. Additionally, we compared the performance of the new risk score with that of the MASCC score, which is the most referenced risk scoring system.

## 2 Methods

### 2.1 Patient Cohort

All consecutive adult patients who presented to the Memorial Sloan Kettering Cancer Center (MSK) ED from January 1, 2016, to December 31, 2020, with NF were included in the derivation phase; whereas, patients meeting the same criteria who visited The University of Texas MD Anderson Cancer Center (MDACC) ED from January 1, 2019, to June 1, 2020, were included in the external validation phase. Both institutional databases were queried to identify all consecutive patients with a chief complaint of fever and laboratory results of neutropenia. The inclusion criteria were (1) an absolute neutrophil count (ANC) lower than 1000 cells/μL or, if ANC data were not available, a white blood cell count of up to 500 cells/μL upon the ED visit; (2) a reported or recorded body temperature ≥38°C during the ED presentation; and (3) receipt of systemic cancer therapy within the previous 12 weeks. Patients younger than 18 years were excluded.

### 2.2 Data Collection and Variable Definitions

Patients’ demographics, clinical and cancer-related characteristics, and variables related to the ED visit (including vital signs and laboratory biomarker levels) and any related hospital admissions were collected. The MASCC score was calculated for all patients. All data were extracted from the institutional data warehouse of each participating site, and no manual chart abstraction took place. For the MASCC score subjective item “burden of illness/symptom severity,” we used the structured ED triage acuity level, based on the Emergency Severity Index (ESI) 5-level scale, as a surrogate. Patients triaged as resuscitation/immediate (ESI 1) or emergent (ESI 2) were classified as having severe symptoms; urgent (ESI 3) as moderate; and less urgent (ESI 4) or nonurgent (ESI 5) as none/mild. As a surrogate for the MASCC criterion of “dehydration requiring IV fluids,” we substituted an elevated serum creatinine above the institution-specific upper limit of normal. The “status at onset of fever” component was considered “outpatient” for all the patients, as they were presenting to the ED. The pharmacy database of both institutions was used to identify systemic cancer therapy regimens. The primary adverse outcome of interest was a composite outcome of intensive care unit (ICU) admission, bacteremia, supplemental oxygen therapy after ED discharge, hospital stay longer than 3 days, or in-hospital death. Outcomes were selected a priori for their clinical relevance and to capture key complications indicative of clinical deterioration in ED presentations of NF.^8^^,^^15^^,^^20^, ^21^, ^22^, ^23^, ^24^, ^25^ Laboratory and biomarker measurements were assigned to low, normal, or high categories based on institution-specific cutoff points for each assay.

### 2.3 Data Analysis

Descriptive statistics were used to summarize the main characteristics of both cohorts, including the age, sex, race, and ethnicity distribution, and baseline clinical characteristics. Variables with missing values were examined using Fisher’s exact test to determine whether there is an association between missingness status and outcome. Clinical variables that did not show a statistically significant association with missingness were included in the following analysis, and missing values were set to normal values. The associations of different clinical variables that are known to be associated with NF outcomes and other clinical variables routinely collected during ED visits with the primary composite adverse outcome were examined using univariate regression models. Variables exhibiting significant associations (*P* < .05) in the univariate analysis were further examined using a 2-directional stepwise logistic regression model to develop the scoring system using the MSK cohort. Final predictors in the regression model were checked for collinearity and independence by calculating variation inflation factors for all predictors in the regression models. The points for the risk score were derived based on the beta coefficients of the variables in the final multivariable model by dividing the β coefficient for each predictor by the smallest beta coefficient of the model and rounding it to the nearest integer. Tenfold cross-validation was used to internally validate the risk score, reporting the mean area under the curve (AUC) value and its 95% CI for the receiver operating characteristic (ROC) curve. The risk score was further applied to the MD Anderson cohort as an external validation step. The AUC values and 95% CIs for the ROC curves were used to examine the performance of the newly developed risk score in the derivation and external validation cohorts, along with the performance of the MASCC score in the derivation cohort. The DeLong test was used to determine whether the AUC values for the derivation and external validation cohorts and the MASCC score differed significantly. To evaluate the calibration of the model, we generated calibration curves for both MSK and MD Anderson cohorts and performed the Hosmer–Lemeshow goodness-of-fit test for both cohorts. Furthermore, patients were assigned to 4 risk groups (low, borderline, intermediate, and high) based on 3 cutoff points of the prediction score. Risk thresholds were empirically set based on predefined criteria corresponding to the risk of major adverse outcomes (in-hospital mortality and ICU admission) in the derivation cohort. Low, borderline, and intermediate risk were defined as the first scores at which observed event rates reached <5%, 5% to <15%, and 15% to 25%, respectively, with high risk including all scores associated with rates ≥25%.

The institutional review boards for MSK and MD Anderson approved this study and granted waivers of informed consent. All statistical analyses were performed using R software for Windows (version 4.4.1). Two–sided *P*-values <.05 were considered indicative of statistical significance.

## 3 Results

### 3.1 Patient Characteristics and Primary Adverse Outcomes

A total of 827 patients fulfilled the inclusion criteria in the derivation MSK cohort, whereas 777 patients met these criteria in the external validation MD Anderson cohort (Table 1). The median ages in the development and validation cohorts were 59 (IQR, 46-68) years and 55 (IQR, 36-68) years, respectively. The sex, race, and ethnicity distributions in the 2 cohorts were comparable (Table 1). An overwhelming majority of the patients (> 98% in both cohorts) presented with either emergent or urgent acuity levels. More than half of the patients in both cohorts had hematologic malignancies (58% for MSK and 62% for MD Anderson).

Regarding the main adverse outcomes, the in-hospital mortality rates were 6.3% in the MSK cohort and 3.3% in the MD Anderson cohort, whereas the ICU admission rates were 12.0% and 11.5%, respectively (Table 2). Regarding the other adverse outcomes in the MSK and MD Anderson cohorts, the prevalence rates for bacteremia were 21.4% and 26.8%, respectively; those for hospitalization for more than 3 days were 73.6% and 77.9%, respectively; and those for oxygen supplementation during hospitalization were 27.4% and 42.0%, respectively. Based on these results, the composite adverse outcome rate was 76.5% for the MSK cohort and 82.1% for the MD Anderson cohort.

### 3.2 Model Development and Internal Validation

**Figure 1:**
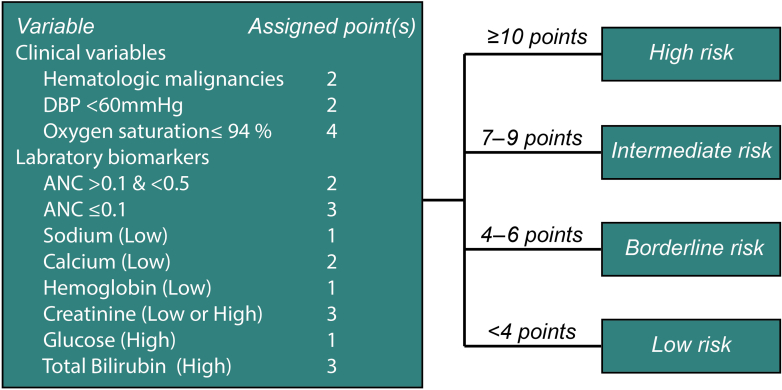
The final NF prognostic scoring system highlights the predictors’ points and the defined risk group thresholds. Low and high laboratory values are based on the institution-specific laboratory reference ranges. DBP, diastolic blood pressure; NF, neutropenic fever.

Table S1 provides a summary of all included candidate predictors and missing-data exclusions stratified by the composite outcome. We performed multivariable logistic regression analysis to develop the primary predictive model (Table S2). The final predictors included in the model were cancer type; initial diastolic blood pressure; initial oxygen saturation; sodium, calcium, creatinine, serum glucose, total bilirubin, and hemoglobin level; and ANC. Figure 1 shows the assigned risk score points for each of these predictors, with a maximum score of 22 points. The median AUC value for the 10-fold cross-internal validation was 0.73 (IQR, 0.69-0.84).

### 3.3 Model Performance, External Validation, and Calibration

**Figure 2:**
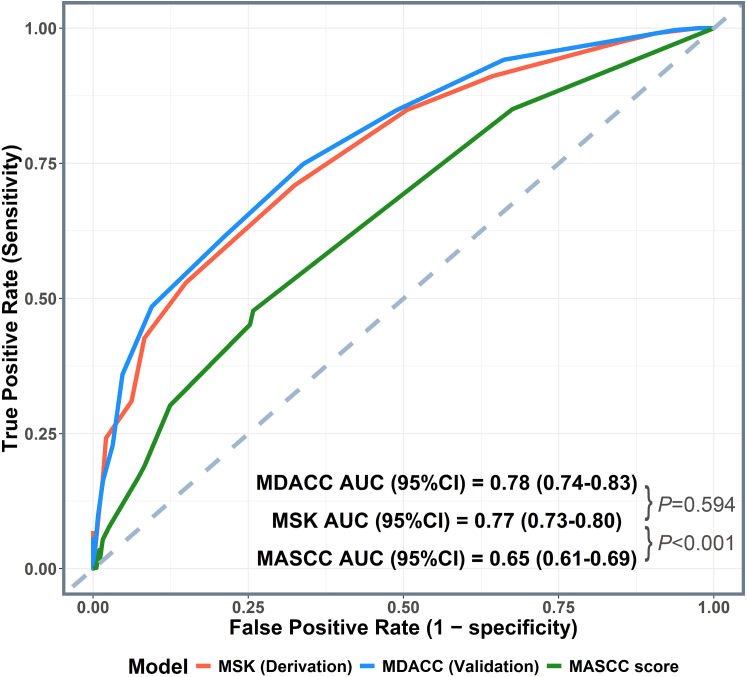
Performance of the newly developed NF risk score for the derivation (MSK) and validation (MD Anderson Cancer Center [MDACC]) cohorts and the MASCC score in predicting the primary composite adverse outcome. MASCC, Multinational Association of Supportive Care in Cancer; MSK, Memorial Sloan Kettering Cancer Center; NF, neutropenic fever.

The AUC value for the ROC curve for the MSK cohort was 0.77 (95% CI, 0.73-0.80), which was not significantly different (*P* = .594) from that for the MD Anderson cohort (0.78 [95% CI, 0.74-0.83]) (Fig 2). However, it was significantly different from the AUC value for the MASCC score (0.65 [95% CI, 0.61-0.69]; *P* < .001). The model’s performance was consistently well-calibrated in both MSK and MD Anderson cohorts, with calibration curves (Fig S1) showing a close alignment between observed and predicted probabilities for both cohorts. A slight deviation at low predicted probabilities was observed in the MD Anderson validation cohort, possibly due to a distribution shift rather than poor calibration, considering the slight difference in baseline characteristics and the adverse outcomes between the MSK and MD Anderson cohorts. The Hosmer-Lemeshow test indicated a good fit in both cohorts (MSK: *X*^*2*^ = 6.64*,* df = 8*, P* = .576*; MD Anderson: X*^*2*^ = 14.29, df = 8*, P* = .074).

### 3.4 Stratification and Risk Groups

**Figure 3:**
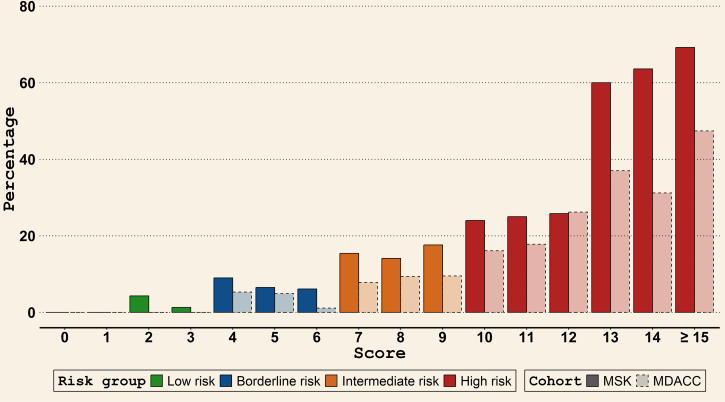
Major adverse outcomes (death or ICU admission) rate for various scores and risk groups in patients presenting with NF in the derivation (MSK) and validation (MD Anderson Cancer Center [MDACC]) cohorts. ICU, intensive care unit; MSK, Memorial Sloan Kettering Cancer Center; NF, neutropenic fever.

Following criteria established a priori (as described in the methods), we stratified the patients into 4 risk categories: low (<4 points), borderline (4-6 points), intermediate (7-9 points), and high (≥10 points) (Fig 1). The rates of different adverse outcomes in these risk groups are listed in Table 3. Among the patients in the MSK and MD Anderson cohorts, 15.1% (125/827) and 4.0% (31/777), respectively, were categorized as low risk, whereas 40.9% (338/827) and 29.2% (227/777) were considered borderline risk. In the MSK and MD Anderson cohorts, low-risk patients had 0% in-hospital mortality, 1.6% and 0% ICU admission, 6.4% and 9.7% bacteremia, and 8.0% and 19.4% required oxygen supplementation, whereas high-risk patients experienced 18.5% and 6.8% in-hospital mortality, 31.8% and 23.7% ICU admission, 41.4% and 39.4% bacteremia, and 60.5% and 57.6% required oxygen supplementation, respectively (Table 3). Figure 3 shows the major adverse outcomes rate (in-hospital mortality and ICU admission rates combined) for each risk group in both cohorts. The major adverse outcomes rate in both cohorts increased incrementally along with the risk score, going from 0% at scores of 0 and 1 to ≥40% for scores >15.

**Table 3:** Performance of the new NF prognostic score: adverse outcomes across risk groups identified by the score (reported as number [%] of cases with the specific adverse outcome)

Outcome	Low risk (< 4 points)		Borderline risk (4-6 points)		Intermediate risk (7-9 points)		High risk (≥ 10 points)	
	MSK (n = 125)	MD Anderson (n = 31)	MSK (n = 338)	MD Anderson (n = 227)	MSK (n = 207)	MD Anderson (n = 283)	MSK (n = 157)	MD Anderson (n = 236)
Major adverse outcomes, n (%)								
In-hospital death	0 (0)	0 (0)	8 (2.4)	2 (0.9)	15 (7.2)	8 (2.8)	29 (18.5)	16 (6.8)
ICU admission	2 (1.6)	0 (0)	19 (5.6)	8 (3.5)	28 (13.5)	25 (8.8)	50 (31.8)	56 (23.7)
Other adverse outcomes, n (%)								
Bacteremia	8 (6.4)	3 (9.7)	45 (13.3)	31 (13.7)	59 (28.5)	81 (28.6)	65 (41.4)	93 (39.4)
Oxygen supplementation	10 (8.0)	6 (19.4)	57 (16.9)	62 (27.3)	65 (31.4)	122 (43.1)	95 (60.5)	136 (57.6)
Hospital stay >3 d	55 (44.0)	11 (35.5)	235 (69.5)	142 (62.6)	176 (85.0)	229 (80.9)	143 (91.1)	223 (94.5)
Composite adverse outcome, n (%)	56 (44.8)	13 (41.9)	242 (71.6)	153 (67.4)	182 (87.9)	243 (85.9)	153 (97.5)	229 (97.0)

ICU, intensive care unit; MSK, Memorial Sloan Kettering Cancer Center; NF, neutropenic fever.

### 3.5 Comparison of the Final Scoring System with the MASCC Score

Table S3 presents a comparison of adverse outcome rates in the low- and high-risk groups for the final scoring system and the MASCC system in the derivation MSK cohort. The major adverse outcome rates differed significantly for the final scoring system and the MASCC score in the low-risk group (1.6% vs 8.8%; *P* = .005), but the rates were comparable in the high-risk group (35.7% vs 38.2%; *P* = .650). The rates for the other adverse outcomes also differed significantly for the final scoring system and the MASCC score in the low-risk group: bacteremia, 6.4% vs 17.8% (*P* = .001); in-hospital oxygen supplementation, 8.0% vs 21.7% (*P* < .001); and hospital stay longer than 3 days, 44.4% vs 74.2% (*P* < .001).

## 4 Limitations

Our study had some limitations. MSK and MD Anderson are both large, urban academic, comprehensive cancer centers with dedicated oncologic EDs, which may not be reflective of general EDs, particularly those in rural and nonacademic institutions. Prospective validation of the developed scoring system in a large number of general EDs and comparison with other risk-stratification models in such a setting are needed. In addition, we did not use the classic definition of NF as outlined by the Infectious Disease Society of America; instead defining NF as a single temperature reading of at least 38 °C and an ANC lower than 1000/mm^3^ at presentation. According to the Infectious Disease Society of America guidelines for NF, “these definitions are not hard and fast rules,” and we deliberately avoided including any variables based on subjective assessments, such as “expected decline to < 500 cells/mL over 48 hours.”^26^ In addition, our study population included only those receiving active systemic cancer treatment, which is similar to the populations used to develop the original MASCC and CISNE scoring systems. This may have skewed our results toward a healthier patient population being included in our study compared to the broad NF population and excluding potentially sicker patients, for example, those too weak to tolerate treatment or for whom treatment is not an option, particularly those with advanced hematological malignancies. Moreover, considering the retrospective nature of our study, we were unable to perform a direct comparison of our risk score with the CISNE score due to the difficulty of accounting for certain variables, specifically, Eastern Cooperative Oncology Group performance status, which is not commonly reported in the ED, and mucositis, which is often not reliably recorded in the ED setting. This may be an avenue for future research. Also, in this study, we defined short hospital length of stay as <72 hours as an objective surrogate for the broad set of inpatient complications described in the MASCC study,^8^ many of which are difficult to measure objectively. Historically, the Centers for Medicare & Medicaid Services (CMS) applied a 72-hour window for bundling outpatient services into an inpatient admission, giving this interval administrative relevance.^27^ In febrile neutropenia, multiple studies likewise use 72 hours as the key early reassessment point, particularly for determining clinical stability and guiding antibiotic de-escalation.^28^^,^^29^ Together, these precedents support a ≤72-hour window as an evidence–based short-term outcome period in NF. However, given that CMS currently uses the Two-Midnight Rule,^30^ and there is no universally accepted definition of a short-term hospital stay, caution is warranted when interpreting a ≤72-hour threshold as a standardized metric. Consideration of an even shorter length-of-stay definition, such as <48 hours, may represent a reasonable avenue for future investigation. Furthermore, whereas our model has considerable room for refinement and improvement in accuracy considering its moderate predictive performance, we aimed for a balance between predictive power and clinical utility, favoring a user-friendly tool over harder-to-implement machine learning techniques that are usually less practical for widespread use (at least for the time being). Finally, our scoring system should be viewed as complementary to, and not as a substitute for, clinical judgment, as physician gestalt retains value beyond the raw data frequently captured by computational models.^31^, ^32^, ^33^, ^34^, ^35^

## 5 Discussion

In this retrospective multi-institutional study, we developed and externally validated a novel prognostic scoring system for patients presenting to the ED who were undergoing active cancer care. This risk-stratification tool compares favorably with the established prognostic MASCC score, with better sensitivity and AUC values. Internal cross-validation of the risk score with similar AUC values in the external validation cohort supports its generalizability.

Our study demonstrated that selected frequently used clinical and laboratory parameters can effectively predict adverse outcomes in our patient population. The variables used in the final predictive model—initial diastolic blood pressure; initial oxygen saturation; sodium, calcium, creatinine, serum glucose, total bilirubin, and hemoglobin level; and ANC—have been validated with other scoring systems (eg, MASCC, CISNE, Acute Physiology and Chronic Health Evaluation II [APACHE II], Sequential Organ Failure Assessment) in both oncologic and nononcologic patient populations and have long been recognized in the oncology literature as prognostic markers of disease severity and outcome.^8^^,^^9^^,^^36^, ^37^, ^38^, ^39^ Hyponatremia, the most common electrolyte disturbance among hospitalized patients, has been consistently associated with increased inpatient mortality in the general population,^36^^,^^37^ with its prognostic effect appearing even more pronounced among patients with metastatic cancer.^40^ Abnormal serum creatinine levels, whether elevated or decreased, have clear prognostic implications. In the APACHE-II Score, both high and low creatinine concentrations correlate with inferior survival,^36^^,^^37^^,^^41^ reflecting the U-shaped association observed in general hospitalized populations, where deviations from physiologic norms are linked to increased inpatient mortality.^42^ In our model, elevated creatinine may also serve as an objective surrogate for the MASCC criterion of dehydration, originally defined as the subjective need for parenteral fluids.^8^ Hyperglycemia carries established prognostic significance. It is incorporated as a variable in the CISNE model,^9^ and has also been linked to increased inpatient mortality in hematologic malignancies, particularly acute leukemia, where elevated glucose levels correlate with higher rates of complicated infections and in-hospital death.^43^

Hypercalcemia remains a clinically meaningful prognostic marker in cancer. In a large national analysis, patients with hypercalcemia of malignancy had more than twice the in-hospital mortality compared with those without hypercalcemia,^44^ underscoring its association with advanced disease and poor short-term outcomes. Elevated bilirubin level has likewise shown prognostic relevance across oncologic populations. Among patients with neutropenia or febrile neutropenia, bilirubin level elevation independently predicts ICU transfer and in-hospital mortality,^45^^,^^46^ reinforcing its value as a short-term prognostic indicator in acute care settings. Anemia (low hemoglobin concentration) independently predicts reduced survival in both solid tumors (lung, head and neck, and prostate) and hematologic cancers.^47^ In the same cohort that identified bilirubin levels as a prognostic factor, Lee et al^46^ also demonstrated that low hemoglobin independently predicted ICU admission and in-hospital mortality, further emphasizing anemia’s importance as a short-term prognostic marker in febrile neutropenia.

Finally, profound ANC depression and the presence of hematologic malignancy are well-established predictors of poor outcomes in febrile neutropenia, as validated in the MASCC risk index.^48^

A key advantage of our scoring system is that it utilizes clinical variables and laboratory biomarkers that are readily available in most EDs, with results frequently obtained within 1 hour of triage. Our model’s data-centered approach, which does not rely on subjective clinical impressions, enables integration with most current electronic medical/health records, with the possibility of autogenerated scores as a clinical decision support system to be used in the ED. This could enable quicker ED disposition decisions, allowing low-risk patients to be discharged more efficiently and high-risk patients to receive accelerated intensive hospital-based care. Improved door-to-physician and door-to-disposition times have been associated with improved outcomes and increased patient satisfaction with care provided during ED visits.^49^

A strength of our predictive risk score is its discrimination ability to identify patients at low risk for major events such as death and ICU admission, the lack of which is a significant limitation of the MASCC score. Although the composite adverse outcome was observed in >40% of low-risk patients of both cohorts, the vast majority were related to hospitalizations longer than 3 days, whereas rates for major adverse events and serious complications, including mortality, ICU admission, and bacteremia, remained low, supporting the low-risk classification. The group categorized as “low risk” may be considered for outpatient management when supported by physician judgment and when the patient meets all of the following criteria: ability to take and tolerate appropriate oral antibiotics, receipt of adequate education, and reliable outpatient follow-up. Patients classified as ‘borderline risk,’ who are unlikely to experience major events but may have adverse outcomes such as requiring oxygen supplementation or a prolonged hospital stay, may be considered for observation or, under limited circumstances, outpatient management. Although researchers have attempted various modifications of the MASCC score to address its low sensitivity,^15^^,^^50^, ^51^, ^52^ we propose our simple scoring system as a promising and practical alternative.

In conclusion, in this large, retrospective, multi-institutional study, we developed and derived a novel, updated prognostic scoring system for patients with NF, demonstrating improved sensitivity and clinical utility when compared with existing models. The use of commonly available objective clinical and laboratory data, coupled with strong internal and external validation, supports its feasibility as a risk-stratification tool in emergency settings while avoiding the need for extensive computing power to use artificial intelligence or machine learning models. Our findings suggest that this tool will aid in clinical decision-making and optimize resource utilization. Future work in prospective multicenter studies in general EDs, along with the implementation of this prognostic scoring system, is warranted.

## Author Contributions

Conceptualization, S.N., A.Q., A.D., S-J.Y.; methodology, S.N., A.Q., A.D.; formal analysis, A.D., A.Q., Z.L.; investigation, S.N., A.Q., A.D., J.S.; resources, S.N., A.Q., K.A., A.K., and S-J.Y.; data curation, S.N., A.Q., A.D., J.S., R.P.J., E.O.; writing—original draft preparation, S.N., A.Q., A.D., A.B., D.Z., A.K., and S-J. Y.; writing—review and editing, S.N., A.Q., A.D., A.B., D.Z., P.C., Z.L., M.K.W., K.A., A.K., and S-J. Y; visualization, A.Q. and A.D.; supervision, S.N., A.K, and S-J. Y.; project administration, S.N and A.Q. All authors have read and agreed to the published version of the manuscript.

## Funding and Support

This research was funded in part through the 10.13039/100000002NIH/NCICancer Center Support Grant P30 CA008748 awarded to Memorial Sloan Kettering Cancer Center and in part by the National Cancer Institute through The University of Texas MD Anderson Cancer Center’s Cancer Center Support Grant (P30CA016672).

## Conflict of Interest

All authors have no conflicts of interest to declare.
