# Supplementary material for: Development and Validation of a Prognostic Risk Score for Patients With Cancer and Neutropenic Fever Presenting to the Emergency Department
Source: J Am Coll Emerg Physicians Open. 2026 Feb 26;7(2):100347. doi: 10.1016/j.acepjo.2026.100347 (PMC12955640; doi:10.1016/j.acepjo.2026.100347)
Supplement: Supplementary Figure 1 and Tables 1-3 [file mmc2.docx]

**SUPPLEMENTAL DATA**

**Supplemental Figures**

**
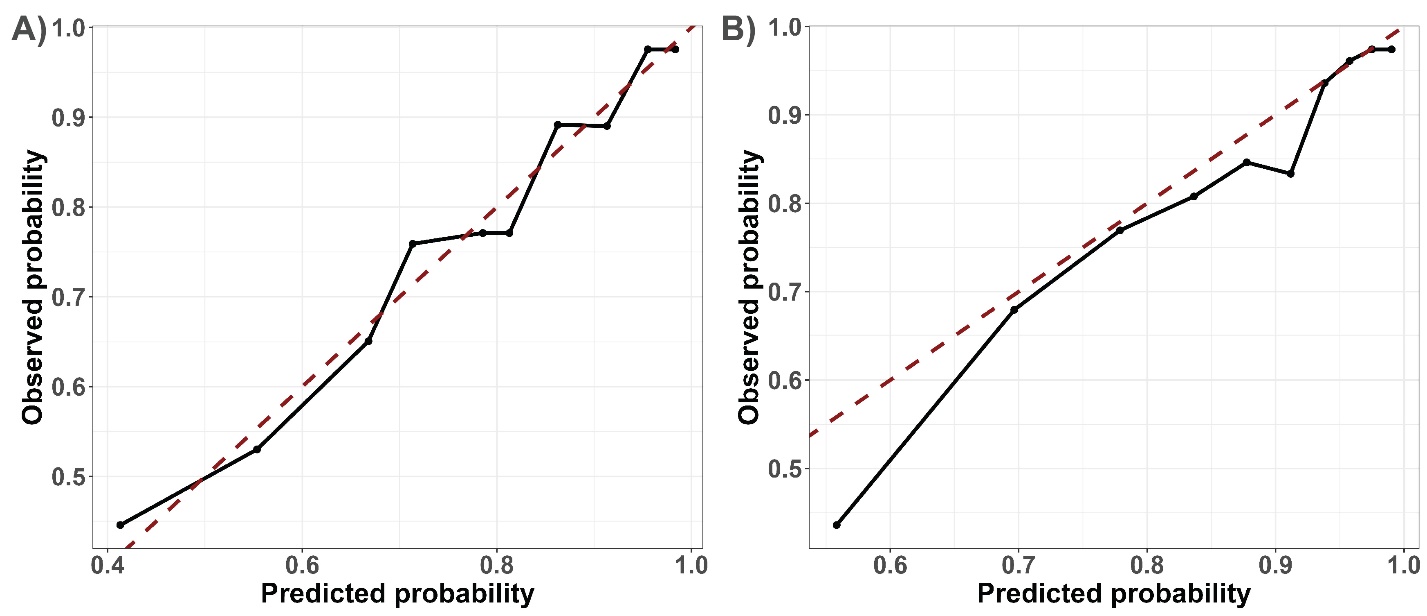
**

**Figure S1. Calibration curves for the study cohorts.** A) Calibration curve for MSK derivation cohort and B) Calibration curve for MD Anderson validation cohort.

**Supplemental table**

**Table S1.** Summary of collected predictors and missing data exclusions stratified by the composite outcome.

| **Variable** | **Composite outcome (Stay > 3 days/ICU/Death/Blood/O2** | | **Assessed for non-informative missingness^1^ / included in further analyses^2^** |
| --- | --- | --- | --- |
|  | **Yes** | **No** |  |
| Age, years |  |  | **-** |
| <40 | 95 (69%) | 42 (31%) |  |
| >65 | 234 (81%) | 56 (19%) |  |
| 40-64 | 304 (76%) | 96 (24%) |  |
| Sex |  |  | **-** |
| Female | 290 (71%) | 116 (29%) |  |
| Male | 343 (81%) | 78 (19%) |  |
| Race |  |  | **-** |
| Asian | 68 (76%) | 22 (24%) |  |
| Black | 54 (68%) | 26 (32%) |  |
| Others or unknown | 71 (81%) | 17 (19%) |  |
| White | 440 (77%) | 129 (23%) |  |
| Smoking |  |  | **-** |
| Cigarette smoker current | 50 (82%) | 11 (18%) |  |
| Never used | 367 (74%) | 126 (26%) |  |
| Previous use | 195 (79%) | 51 (21%) |  |
| Unknown | 15 (83%) | 3 (17%) |  |
| Acuity/ESI |  |  | **-** |
| Resuscitation | 11 (100%) | 0 (0%) |  |
| Emergent | 354 (80%) | 86 (20%) |  |
| Urgent | 267 (71%) | 108 (29%) |  |
| Unknown | 1 (100%) | 0 (0%) |  |
| CCI |  |  | **-** |
| <6 | 134 (72%) | 51 (28%) |  |
| >20 | 163 (80%) | 40 (20%) |  |
| 10-20 | 168 (73%) | 61 (27%) |  |
| 6-10 | 140 (80%) | 35 (20%) |  |
| Missing | 28 (80%) | 7 (20%) |  |
| COPD |  |  | **-** |
| No | 588 (76%) | 188 (24%) |  |
| Yes | 45 (88%) | 6 (12%) |  |
| Fungal |  |  | **-** |
| No | 625 (76%) | 194 (24%) |  |
| Yes | 8 (100%) | 0 (0%) |  |
| Cancer Type |  |  | **-** |
| Solid tumor | 232 (67%) | 116 (33%) |  |
| Hematologic | 401 (84%) | 78 (16%) |  |
| Diastolic blood pressure |  |  | **-** |
| ≥60 mmHg | 521 (74%) | 180 (26%) |  |
| <60 mmHg | 112 (89%) | 14 (11%) |  |
| Systolic blood pressure |  |  | **-** |
| ≤90 mmHg | 35 (85%) | 6 (15%) |  |
| >90 mmHg | 598 (76%) | 188 (24%) |  |
| Pulse, beats per minute (BPM) |  |  | **-** |
| ≤90 BPM | 113 (80%) | 28 (20%) |  |
| >90 BPM | 520 (76%) | 166 (24%) |  |
| Oxygen saturation |  |  | **-** |
| >94 % | 548 (74%) | 189 (26%) |  |
| ≤94 % | 85 (94%) | 5 (6%) |  |
| Temperature |  |  | **-** |
| <38 °C | 144 (79%) | 38 (21%) |  |
| 38-38.3 °C | 135 (69%) | 60 (31%) |  |
| 38.3-38.95 °C | 189 (78%) | 54 (22%) |  |
| ≥38.95 °C | 165 (80%) | 42 (20%) |  |
| Sodium level^3^ |  |  | **-** |
| Normal | 438 (74%) | 151 (26%) |  |
| Low | 190 (82%) | 41 (18%) |  |
| Potassium level |  |  | **-** |
| Normal | 552 (76%) | 175 (24%) |  |
| High | 8 (89%) | 1 (11%) |  |
| Low | 58 (85%) | 10 (15%) |  |
| Chloride level |  |  | **-** |
| Normal | 508 (75%) | 168 (25%) |  |
| High | 16 (84%) | 3 (16%) |  |
| Low | 104 (83%) | 21 (17%) |  |
| Carbon dioxide level^3^ |  |  | **-** |
| Normal | 604 (76%) | 190 (24%) |  |
| Low | 25 (93%) | 2 (7%) |  |
| Blood urea nitrogen |  |  | **-** |
| Normal | 463 (73%) | 169 (27%) |  |
| High | 153 (88%) | 20 (12%) |  |
| Low | 13 (81%) | 3 (19%) |  |
| Creatinine level |  |  | **-** |
| Normal | 525 (74%) | 180 (26%) |  |
| High | 88 (90%) | 10 (10%) |  |
| Low | 15 (88%) | 2 (12%) |  |
| Glucose level^4^ |  |  | **-** |
| Normal | 490 (75%) | 166 (25%) |  |
| High | 137 (84%) | 26 (16%) |  |
| Calcium level^3^ |  |  | **-** |
| Normal | 429 (72%) | 163 (28%) |  |
| Low | 195 (88%) | 26 (12%) |  |
| Magnesium level^3^ |  |  | Yes / Yes |
| Normal | 151 (77%) | 44 (23%) |  |
| Low | 81 (85%) | 14 (15%) |  |
| Missing | 401 (75%) | 136 (25%) |  |
| Albumin level^3^ |  |  | Yes / Yes |
| Normal | 114 (67%) | 55 (33%) |  |
| Low | 281 (81%) | 68 (19%) |  |
| Missing | 238 (77%) | 71 (23%) |  |
| Aspartate aminotransferase level^4^ |  |  | Yes / Yes |
| Normal | 312 (76%) | 100 (24%) |  |
| High | 74 (80%) | 19 (20%) |  |
| Missing | 247 (77%) | 75 (23%) |  |
| Alanine aminotransferase level^4^ |  |  | Yes / Yes |
| Normal | 321 (75%) | 106 (25%) |  |
| High | 73 (81%) | 17 (19%) |  |
| Missing | 239 (77%) | 71 (23%) |  |
| Alkaline phosphatase level^4^ |  |  | Yes / Yes |
| Normal | 313 (77%) | 95 (23%) |  |
| High | 81 (74%) | 28 (26%) |  |
| Missing | 239 (77%) | 71 (23%) |  |
| Bilirubin total level^4^ |  |  | Yes / Yes |
| Normal | 293 (72%) | 116 (28%) |  |
| High | 101 (94%) | 7 (6%) |  |
| Missing | 239 (77%) | 71 (23%) |  |
| Bilirubin direct^4^ |  |  | Yes / Yes |
| Normal | 226 (71%) | 91 (29%) |  |
| High | 89 (94%) | 6 (6%) |  |
| Missing | 318 (77%) | 97 (23%) |  |
| Anion gap |  |  | - |
| Normal | 543 (76%) | 167 (24%) |  |
| High | 9 (100%) | 0 (0%) |  |
| Low | 70 (76%) | 22 (24%) |  |
| White blood cell count |  |  | - |
| Normal | 8 (89%) | 1 (11%) |  |
| High | 15 (94%) | 1 (6%) |  |
| Low | 610 (76%) | 192 (24%) |  |
| Hemoglobin^3^ |  |  |  |
| Normal | 43 (60%) | 29 (40%) |  |
| Low | 590 (78%) | 165 (22%) |  |
| Platelet count^3^ |  |  |  |
| Normal | 103 (66%) | 52 (34%) |  |
| Low | 530 (79%) | 142 (21%) |  |
| International Normalized Ratio |  |  | Yes / No |
| Not missing | 329 (80%) | 81 (20%) |  |
| Missing | 304 (73%) | 113 (27%) |  |
| PT |  |  | Yes / No |
| Not missing | 326 (80%) | 81 (20%) |  |
| Missing | 307 (73%) | 113 (27%) |  |
| PTT |  |  | Yes / No |
| Not missing | 329 (80%) | 80 (20%) |  |
| Missing | 304 (73%) | 114 (27%) |  |
| Lactic acid |  |  | Yes / Yes |
| Normal | 234 (76%) | 75 (24%) |  |
| High | 314 (77%) | 95 (23%) |  |
| Procalcitonin |  |  | Yes / No |
| Not missing | 228 (85%) | 40 (15%) |  |
| Missing | 405 (72%) | 154 (28%) |  |
| CRP |  |  | Yes / Yes |
| High | 11 (92%) | 1 (8%) |  |
| Missing | 622 (76%) | 193 (24%) |  |
| ESR |  |  | Yes / Yes |
| Normal | 1 (100%) | 0 (0%) |  |
| High | 1 (50%) | 1 (50%) |  |
| Missing | 631 (77%) | 193 (23%) |  |
| LDH |  |  | Yes / Yes |
| High | 2 (100%) | 0 (0%) |  |
| Missing | 631 (76%) | 194 (24%) |  |
| Troponin |  |  | Yes / No |
| Not missing | 73 (87%) | 11 (13%) |  |
| Missing | 560 (75%) | 183 (25%) |  |

**Abbreviations:** CCI, Charlson Comorbidity Index, COPD, chronic obstructive lung disease.

^1^Fihser’s exact test used to assess association between outcome missingness. ^2^Variable is selected for further analysis if p-value<0.05 and missing values were set to normal levels. ^3^Values with high levels were set to normal as percentage of patients with high values was below 1%. ^4^Values with low levels were set to normal as percentage of patients with low values was below 1%

**Table S2.** Final risk score model predictors and assigned points.

| **Variable** | **Beta coefficient** | **Standard error of the coefficient** | **Assigned points** |
| --- | --- | --- | --- |
| Hematologic cancer | 0.7818 | 0.1913 | 2 |
| Initial diastolic blood pressure <60 mmHg | 0.7441 | 0.3218 | 2 |
| Initial pulse oxygen saturation ≤94% | 1.7613 | 0.4883 | 4 |
| Absolute neutrophil count >0.1 and <0.5 K/μL | 0.6500 | 0.2422 | 2 |
| Absolute neutrophil count ≤0.1 K/μL | 1.3874 | 0.2757 | 3 |
| Sodium - low | 0.4249 | 0.2235 | 1 |
| Calcium - low | 0.7125 | 0.2490 | 2 |
| Hemoglobin - low | 0.5325 | 0.2906 | 1 |
| Creatinine - low | 1.0388 | 0.7943 | 2 |
| Creatinine - high | 1.0002 | 0.3702 | 2 |
| Serum glucose - high | 0.4360 | 0.2581 | 1 |
| Total bilirubin - high | 1.3222 | 0.4163 | 3 |

**Table S3.** Comparison of the MASCC and final risk score groups with different outcomes in the derivation model (reported as the percentage of cases with the specific outcome).

| **Outcome** | **Final risk score** | | **MASCC** | |
| --- | --- | --- | --- | --- |
|  | **Low risk**  **(<4 points)** | **High risk**  **(≥10 points)** | **Low risk**  **(≥21 points)** | **High risk**  **(<21 points)** |
| Major outcomes | 1.6 | 35.7 | 8.8 | 38.2 |
| In-hospital death | 0.0 | 18.5 | 3.3 | 21.3 |
| ICU admission | 1.6 | 31.8 | 8.0 | 32.4 |
| Other outcomes |  |  |  |  |
| Positive blood culture | 6.4 | 41.4 | 17.8 | 39.7 |
| Oxygen supplementation | 8.0 | 60.5 | 21.7 | 56.6 |
| Hospital stay >3 days | 44.4 | 91.7 | 72.1 | 83.7 |
| Composite outcome | 44.8 | 97.5 | 74.2 | 88.2 |
